# The fellow effect on college students’ academic performance

**DOI:** 10.3389/fpsyg.2022.1055963

**Published:** 2022-12-22

**Authors:** Guohua Zeng, Minglong Zhong, Wenwen Wu

**Affiliations:** School of Economics and Management, Jiangxi University of Science and Technology, Ganzhou, Jiangxi, China

**Keywords:** peer effect, fellow effect, academic achievement, university students, university education, gender differences

## Abstract

This paper uses data from the 2018 College Graduates Employment Survey in a province in central China to investigate whether there is a fellow effect (a special kind of peer effect) among groups of college students in colleges and universities. It was found that a group of fellows with higher academic achievement would have a significant positive effect on individual students’ achievement; conversely, it would have a significant negative effect on individual student’s achievement. To avoid endogeneity problems, this paper conducted a two-stage regression analysis using the average education level of the parents of the fellow as an instrumental variable; to ensure the robustness of the findings, this paper used the fellow sample at the municipal level for the regression. The analysis of heterogeneity found that the effect of good grades in the fellow had a greater impact on the individual academic performance of girls compared to boys; in terms of geography, the effect of fellow showed a decreasing trend from eastern to central and western China; in terms of major categories, the effect of fellow also showed a greater difference between humanities majors and social science majors.

## Introduction

School is the most important place for adolescents to be socialized outside of the home, and the peer group in school is closely related to adolescent development. Coleman (1966) argues that the peer group in school has as much influence on adolescent development as important factors such as parental involvement, quality of education, and class size (Sacerdote, 2011). Numerous empirical studies have shown that the better the peer group at school or in the classroom, the better the academic performance of the individual (Wu and Zhang, 2020; Xuesong, 2020; Yang and Huang, 2020). The “one who stays nearer vermilion gets stained red” effect. According to sociological reference group theory, peer groups may have a “social contract “function (Kelley, 1952), i.e., peers may compare themselves with each other due to the “social contract” function (Kelley, 1952). The “discouraging” effect is caused by the comparison of peers with each other, which leads to a decrease in self-conception or self-esteem of the individual who is in a group of superior peers. The above research supports the notion that those who are close to the vermilion are red and those who are close to the ink are black.

The special group of peers, the fellow, is defined in the Dictionary as people who come from the same region or the same province. Zhong and Qian (2017) believe that due to “carrying” common perceptions, cultural characters, and regional psychology, the fellow effect is mediated by the perception of fellow, which in turn leads to specific fellow behavior. Zhang and Jiang (2013) through their research, they argue that migrant workers in different places will form mutual help fellow associations; (Shigen, 2019; Liu and Ma, 2022) on the other hand, found that fellow relationships have a significant impact on the internal control and financial risk of companies. College students in higher education come from all over the world, and when individual students enter a brand new environment, they tend to look for people or organizations that come from the same place and have the same language symbols as they do to communicate. So, do fellow relationships among students in higher education have an effect on students’ academic performance? Exploring the study of the fellow effect in higher education can further enrich the depth of peer effect research and provide a theoretical basis for understanding the academic achievement choices of higher education students.

## Review of the literature

Research on peer effects in education began in the 1960s when Coleman (1966) conducted a study on the effects of peers on student achievement. Since then many scholars have conducted research, for example, Bruce (2001) used random assignment data from freshman dorm rooms at Dartmouth College to examine peer effects among college roommates and showed that peers had a significant effect on average academic performance and membership in clubs. Eisenberg et al. (2014) argue that the gender composition of university peers may have peer effects on risky behavior, which in turn may affect academic performance. Isabel Pessoa de Arruda Raposo (2018) and Raposo and Gonalves (2018) found that the more peers in the classroom, the better the students’ average performance; in terms of the assessment of math and Portuguese performance, a one standard deviation increase in peer performance would increase individual student performance by 30% of the standard deviation in a diffusion study of students’ academic performance in Brazilian public schools. Diemer (2022) research findings on endogenous peer effects in friendship networks suggest that the interactive friendship networks between children of foreign origin and immigrant children show greater peer effects and will widen the gap in academic achievement between native and immigrant children.

Related studies in China started late, mostly based on classes and dormitories to develop the analysis of peer effects on academic performance. For example, Xiaojuan (2015) found that the peer effect on academic achievement at the class level was greater than the peer effect at the dormitory level, and that the peer effect not only had gender differences, but also differences in learning ability were affected differently by the peer effect. Yaoming and Qinying (2017) also reached similar conclusions in their study of undergraduate dormitory-level peer effects, concluding that dormitory peer effects have a significant positive impact on individual achievement, with large differences in the impact of peer effects on individuals with different learning abilities. Zhimei (2020) used questionnaire data from several different levels of universities to classify students’ academic performance into positive learning benefits and negative learning behaviors, and to classify roommate relationships into “joint progress,” “negative interference” and “subtle influence.” The results showed that the peer effects at the dormitory and class levels had significant positive and negative effects on academic performance, and there were heterogeneous differences by gender. Ma and Huang (2021) used two undergraduate students from a “double first-class” university as the subjects, the study was conducted to overcome the selection bias in the study of peer effects by randomly assigning them to dormitories, and to investigate peer effects in four aspects: the average peer effect of roommates and the trend of change, the heterogeneity of peer effects, the structural differences of peer effects, and the competitiveness of peer effects. and the competitive nature of the peer effect. The study found that college roommate performance had a positive peer effect on peers and that the heterogeneity of the peer effect existed between roommate academic grades and between genders.

Further, Manski (1993) argues that self-selection problems, reflexive problems (imaging problems), and co-occurrence problems can lead to inaccurate estimates of the peer effect. Self-selection problems, where individuals selectively engage or interact with certain types of people based on their own interests or unobservable hidden factors, are likely to result in peer effects due to “self-selection problems.” Reflexivity refers to the fact that peer groups can have an impact on individual students, while individuals can also have an impact on peer groups, leading to a two-way causal problem in the empirical process. The problem of co-occurrence refers to the fact that students in a class learning and living in the same environment will result in highly correlated learning behavior outcomes, which may be due to the influence of the level of teaching by the teacher in the class. To address the problem of self-selection, some scholars have used randomized and naturalistic experiments to address the problem of self-selection. For example, Bruce (2001) conducted a natural experiment of random assignment of dormitory arrangements for freshmen at Dartmouth in the United States to study peer effects at the dormitory level at that school. To address the reflexive problem, most scholars use instrumental variable methods to address the two-way causality problem in benchmark regressions, such as Qiang (2019) and Zhouhang et al. (2018).

The fellow effect is a special case of the peer effect, which refers to the positive tendency of an individual in a foreign place, when faced with an unfamiliar environment, to have a positive feeling toward people from the same area (city) because they “hold” the same language, culture, emotions, lifestyle, values, religious practices, etc. as he or she does (Jiang et al., 2012). This is a positive emotional involvement and tendency to converge (Jiang et al., 2012). This is a positive emotional involvement and tendency to converge in one’s mind Jiang et al. (2012), which will eventually influence one’s behavior. According to regional psychology, the division of administrative and geographical regions creates different geographical and cultural perceptions, which in turn form the cultural character of a particular region, and the cultural character of different regions ultimately determines regional psychological differences (Zhang and Jiang, 2010). Guo and Zhang (2022) from a cultural psychology perspective, suggest that culture is a potential factor that affects individual behavior all the time. When a group lives in the same place for a long time, the group will be potentially influenced by the local social environment and cultural traditions, and only when they interact with people who are influenced by different cultures can they discover the influence of their own culture. Similarly, Zhang and Jiang (2010), in a preliminary construction of the psychological mechanisms of the fellow perception, argue that individuals will only emotionally construct the fellow perception and thus the fellow psychological perception when they are in a different place, and that cultural and environmental differences will cause behavioral differences. Bramoullé et al. (2020) compares the literature on peer effects in networks and the identification of peer effects and argues that The key to studying peer effects lies in the analysis of theoretical models of network interaction. Therefore, this paper analyses the fellow effect (a special kind of peer effect) among university students based on the new research perspective of social networks of fellow groups.

In summary, there are many studies on peer effects in higher education, but there are still several shortcomings: (1) Most of the literature focuses on peer effects at the classroom and dormitory levels, but few scholars have conducted in-depth studies on peer effects at the level of fellow students in the same school and in the same major. (2) Some of the studies only examine the impact of average peer behavior and output on individual students, and fail to distinguish whether and how the heterogeneity of peer behavior or output affects individual students differently. (3) Due to the differences in the selected research subjects and sample data, there is no consensus on the mechanism of peer effects and the mechanism of action.

Therefore, this paper analyzes the fellow effect of college student’s academic performance based on the employment survey data of college graduates in a central province in 2018. As the research object of this paper is the peer effect (fellows effect) of the fellows group of the same school and the same major, the fellow group is formed through the random assignment mechanism of the college entrance examination. Therefore, the self-selection problem causes very little impact on the peer effect (fellows effect) estimates in this paper. To mitigate the reflexive problem, this paper draws on Yuan Zhouhang’s practice of selecting the average education level of the parents of the fellows as an instrumental variable for the proportion of fellows in each achievement level; in addition, this paper draws on (Xuesong, 2020)’s approach, the co-temporality problem is mitigated by controlling for variables such as the province, school, and major from which individuals come.

## Study design

### Model setting

Drawing on Qiang (2019), the following model is used in this paper to estimate the fellow effect on the academic performance of college and university students.


(1) $$Y_{\mathrm{ijk}}=\beta_{0}+\beta_{1}Z_{ijp}+\beta_{2}V{\mathrm{ar}}_{\mathrm{ijp}}+\lambda X_{ijk}+\varepsilon_{i}$$

Dependent variable $Y_{\mathrm{ijk}}$: The overall academic performance rank of student k in major j at school i in that major, divided into five ranks in total, where 1–5 denote overall performance ranking in the bottom 20%, bottom 21–40%, 40–60%, top 21–40% and top 20% of the major, respectively.

Core explanatory variables $Z_{ijp}$: Drawing on Diemer (2022), the percentage of all fellow (provincial) with achievement levels 1, 2, 3, 4 and 5 were used as explanatory variables, respectively: $Z_{\mathrm{ijp}}=\frac{m_{i}}{n}$ (i = 1,2,3,4,5), where n denotes the number of all provincial old villagers for an individual student, and $m_{i}$ denotes the number of fellow with academic achievement level i. $\beta_{1}$ measure the effect of each academic performance level of fellow on the individual student’s performance level.

Control variables$X_{\mathrm{ijk}}$: include individual student’s province of birth, school, type of major, gender, whether they are poor, whether they were born in an urban area, political affiliation, type of second-degree minor, type of club participation, type of parent’s establishment, parent’s education level, help from parent’s family background.

$Var_{ijp}$: denotes the variance of all fellow grades for individual students. That is,$Var_{ijp}=\frac{1}{n}\overset{n}{\underset{i=1}{\sum }}{\left(x_{i}-\bar{x}\right)}^{2}$, $\;\beta_{2}$ measures the effect of the variance of fellow grades on individual student performance.

### Description of data

This paper uses data from the 2018 Employment Survey of College Graduates in a central province of China. The survey was organized and implemented by the Employment Office of the Provincial Education Department and covered all higher education institutions in the province. As some of the majors in some colleges and universities have only one student enrolled in a particular province, resulting in no fellow for that individual student in that school or major. Therefore, data on individual students without fellows were excluded from the sample, and the valid sample size was 179,588 entries. In this data, 128 items of personal information of the individual student are included, such as: ID number, place of birth, school attended, major attended, education level of the student’s parents, etc. The following data (variables) are used in this paper: student’s ID number, overall grade ranking located in this major, provincial code, municipal code, school code, major code attended, gender, category of poor student, whether born in an urban area, political appearance, category of second degree minor subject, type of association attended, type of parent’s unit, parent’s education level, help from parents and family background, whether a teacher-training student, core explanatory variables $Z_{ijp}$ (number of old folks with achievement levels 1–5, respectively, as a percentage of the total number of old folks (provincial or municipal)), variance of fellow’s achievement, and education level of fellow’s parents. In particular, in terms of the identification of the fellows and the number of fellows. Firstly, using the four variables of the provincial code (municipal code), the code of the school attended, the code of the major attended, and the overall grade rank located in the major of all students; then writing python scripts to determine whether students other than student i were born in the same province (city) as a student i and whether they attended the same school and a major; finally, identify the fellow, calculate the core explanatory variables $Z_{ijp}$ and the variance of fellow grades. The main variables are described in detail in Table 1 below.

**Table 1:** Description of variables.

Dependent variable	Academic Performance Level	Overall ranking in the major: 1 = bottom 20%, 2 = bottom 21–40%, 3 = top 40–60%, 4 = top 21–40%, 5 = top 20%
Core explanatory variables	$Z_{ijp}$	$Z_{\mathrm{ijp}}=\frac{m_{i}}{n}$(i = 1,2,3,4,5), where n denotes the number of all provincial fellows for an individual student, and$m_{i}$ denotes the number of fellows with academic achievement level i.
Control variables	The variance of the Old Country results	$Var_{ijp}=\frac{1}{n}\overset{n}{\underset{i=1}{\sum }}{\left(x_{i}-\bar{x}\right)}^{2}$ to measure the degree of volatility in the achievements of the Old Country Group
	Province	The sample contains a total of 31 provinces
	City	The sample contains a total of 360 cities
	School	The sample covers all higher education institutions in the province
	Type of profession	The sample contains a total of 687 sub-disciplines
	Gender	0 = Female, 1 = Male
	Needy Student Category	0 = non-disadvantaged students, 1 = disadvantaged students
	Born in an urban area or not	0 = born in a rural area, 1 = born in an urban area
	Political affiliation	0 = mass, 1 = Communist Youth League member, 2 = preparatory member, 3 = party member
	Second Degree Minor Subject Categories	0 = No; 1 = Yes, Philosophy, 2 = Yes, Economics, 3 = Yes, Law, 4 = Yes, Education, 5 = Yes, Literature, 6 = Yes, History, 7 = Yes, Science, 8 = Yes, Engineering, 9 = Yes, Agriculture, 10 = Yes, Medicine, 11 = Yes, Military, 12 = Yes, Management, 13 = Yes, Art
	Type of association attended	0 = never attended, 1 = faith-based, 2 = academic, 3 = literary, 4 = sports, 5 = technology, 6 = service, 7 = practice, 8 = association, 9 = career, 10 = entrepreneurial, 11 = other
	Parental literacy	0 = never attended school, 1 = junior high school and below, 2 = high school, 3 = college, 4 = bachelor’s degree, 5 = graduate
	Help from parents and family background	1 = largely unhelpful, 2 = yes, but not very helpful, 3 = yes, moderately helpful, 4 = yes, more helpful, 5 = yes, very helpful
	Whether you are a teacher trainee	0 = non-teaching students, 1 = teaching students

### Descriptive statistics

Table 2 shows the descriptive statistics of the variables. It can be seen that the overall grade ranking is located in the major with a maximum value of 5, a minimum value of 1 and a mean value of 3.682. The number of individual student peers varies considerably with a minimum value of 1, a maximum value of 2,308, and a standard deviation of 339.9 due to the wide variation in admissions policies across the provinces. For the core explanatory variables, the means of each grade of individual student provincial peer scores as a proportion of all peers were 0.045, 0.115, 0.271, 0.251 and 0.381, respectively, with standard deviations of 0.077, 0.115, 0.159, 0.150 and 0.177; the mean value of the variance of provincial fellow scores was 1.153. In terms of individual student characteristics, the numbers of male, the number of females were about the same, the number of poor students, the number of those born in urban areas, and the number of teacher-training students were relatively small, and the mean value of political affiliation was 0.984, with most students’ political affiliation being that of a member of the Communist Youth League. The mean value of parents’ education level was 1.421, with a standard deviation of 0.848, indicating that the majority of students’ parents have a low level of education, mainly at the junior to senior secondary level. The parents and family backgrounds are low in helping students in various areas, with a mean value of 1.951.

**Table 2:** Descriptive statistics (provincial).

Main variables		Sample size	Average	Minimum value	Maximum value	Standard deviation
Overall ranking in the program		179,588	3.682	1	5	1.164
Number of individual student provincial fellows		179,588	166.5	1	2,308	339.9
Individual student provincial fellow score rating of i as a proportion of all fellows	i = 1	179,588	0.045	0	1	0.077
	i = 2	179,588	0.115	0	1	0.115
	i = 3	179,588	0.271	0	1	0.159
	i = 4	179,588	0.251	0	1	0.150
	i = 5	179,588	0.318	0	1	0.177
The variance of provincial fellows ‘results		179,588	1.153	0	4	0.502
Gender		179,588	0.5	0	1	0.50
Whether you are a needy student		179,588	0.135	0	1	0.342
Born in an urban area or not		179,588	0.266	0	1	0.442
Political affiliation		179,588	0.984	0	3	0.413
Second Degree Minor Subject Categories		179,588	0.686	0	13	2.353
Type of association attended		179,588	4.284	0	11	3.242
Parental literacy		179,588	1.421	0	5	0.848
Help from parents and family background		179,588	1.951	1	5	1.138
Whether a student majoring in normal education		179,588	0.107	0	1	0.309

## Empirical analysis

### Regression results of the old country effect

Table 3 reports the OLS regression results for the effect of the fellow effect on individual student academic performance in colleges and universities. Columns (1)–(5) denote the number of individual students with a provincial fellow score rating of 1–5 as a percentage of all fellows as the core explanatory variable, i.e., the fellow effect studied in this paper, respectively, to examine its effect on the overall grade ranking of individual students. All of the above regressions control for individual students’ province of birth, school of study, and major, as well as individual student’s type of club participation and type of second-degree minor discipline.

**Table 3:** Regression results for the effect of the fellow effect on academic performance.

		Individual overall student performance ranking in the major				
		(1)	(2)	(3)	(4)	(5)
Number of individual students with a provincial fellow score rating of i as a percentage of all fellows	i = 1	−0.181*** (0.040)				
	i = 2		−0.153*** (0.025)			
	i = 3			−0.106*** (0.018)		
	i = 4				0.037** (0.019)	
	i = 5					0.163*** (0.016)
The variance of the fellow’s results		0.021*** (0.007)	0.016** (0.006)	0.004 (0.006)	0.012* (0.007)	0.007 (0.006)
Gender		−0.455*** (0.007)	−0.455*** (0.007)	−0.455*** (0.007)	−0.455*** (0.007)	−0.455*** (0.007)
Political affiliation		0.381*** (0.006)	0.381*** (0.006)	0.381*** (0.006)	0.381*** (0.006)	0.381*** (0.006)
Whether you are a needy student		0.241*** (0.008)	0.241*** (0.008)	0.240*** (0.008)	0.241*** (0.008)	0.241*** (0.008)
Born in an urban area or not		0.017*** (0.006)	0.017*** (0.006)	0.017*** (0.006)	0.017*** (0.006)	0.017*** (0.006)
Whether a student majoring in normal education		−0.114*** (0.029)	−0.113*** (0.029)	−0.113*** (0.029)	−0.115*** (0.029)	−0.109*** (0.029)
Parental literacy		0.001 (0.00a3)	0.001 (0.003)	0.001 (0.003)	0.001 (0.003)	0.001 (0.003)
Help from parents and family background		−0.001 (0.002)	−0.001 (0.002)	−0.001 (0.002)	−0.001 (0.002)	−0.001 (0.002)
Province		Control	Control	Control	Control	Control
School		Control	Control	Control	Control	Control
Specialities		Control	Control	Control	Control	Control
Type of association attended		Control	Control	Control	Control	Control
Second Degree Minor Subject Categories		Control	Control	Control	Control	Control
_cons		2.552*** (0.275)	2.566*** (0.275)	2.588*** (0.289)	2.519*** (0.280)	2.580*** (0.275)
*R*2		0.099	0.099	0.099	0.098	0.099
*N*		179588.00	179588.00	179588.00	179588.00	179588.00

(1) Figures in brackets are standard errors; (2) ***, ** and * represent 1, 5 and 10% significance levels respectively; (3) The above regression results are all ROBUST regressions.

The results show that (1) when the individual student’s provincial fellow grades were 1, 2 and 3 (lower than the mean of the total sample grade of 3.682), the effect on the individual student’s overall grade rank was significantly negative, and when the core explanatory variable$Z_{ijp}$ (the number of provincial fellow grades of 1, 2 and 3 as a percentage of the total number of fellow grades) increased by 1%, the individual student’s overall grade rank in the major decreased by 0.181, 0.153 and 0.106, respectively. (2) The effect on the student’s overall ranking was significantly positive when the individual student’s provincial fellow achievement level was 4 and 5, respectively, and the student’s overall ranking improved by 0.037 and 0.163 for each 1% increase in the proportion of fellow students in the two achievement levels.

In terms of control variables, there was a significant positive effect of the variance of fellow achievement on individual student achievement, meaning that the greater the dispersion of fellow achievement, the higher the individual student achievement ranking. There was a significant negative effect of the fellow effect on the gender variable, which is consistent with the results of scholars such as Xiaojuan (2015) and Yang and Huang (2020) in their studies of peer effects. Political affiliation, being a poor student or not, and being born in an urban area all had significant positive effects on individual student academic achievement, and being a teacher-training student or not had a significant negative effect on individual student academic achievement. Parental literacy, parental and parental and family background assistance did not have a significant effect on individual student academic achievement.

### Heterogeneity analysis

#### Gender differences in the fellow effect

Table 4 shows that for both males and females, there is a significant negative effect on individual student achievement when the fellow composite ranking rank is at 1, 2 and 3, respectively. When the ranking level of the fellow is 4, there is no significant effect of the male fellow on the individual male student’s performance, while there is a significant positive effect of the female fellow on the individual female student’s performance. There is a significant positive effect on the individual student’s overall grade when the overall grade ranking of the fellow is at 5.

**Table 4:** Regression results of the ranking of individual students’ overall performance by gender fellow.

	Male					Women				
	Overall ranking in the program					Overall ranking in the program				
	(1)	(2)	(3)	(4)	(5)	(6)	(7)	(8)	(9)	(10)
z1	−0.228*** (0.058)					−0.140*** (0.053)				
z2		−0.113*** (0.035)					−0.186*** (0.034)			
z3			−0.077*** (0.026)					−0.126*** (0.025)		
z4				0.029 (0.027)					0.043* (0.025)	
z5					0.135*** (0.024)					0.182*** (0.023)
Var	0.031*** (0.010)	0.019** (0.010)	0.011 (0.010)	0.017* (0.010)	0.013 (0.009)	0.018* (0.009)	0.017* (0.009)	0.003 (0.009)	0.012 (0.009)	0.006 (0.009)
x1	0.399*** (0.009)	0.399*** (0.009)	0.399*** (0.009)	0.399*** (0.009)	0.399*** (0.009)	0.360*** (0.009)	0.361*** (0.009)	0.360*** (0.009)	0.360*** (0.009)	0.360*** (0.009)
x2	0.293*** (0.012)	0.293*** (0.012)	0.293*** (0.012)	0.293*** (0.012)	0.293*** (0.012)	0.186*** (0.011)	0.186*** (0.011)	0.186*** (0.011)	0.186*** (0.011)	0.186*** (0.011)
x3	0.031*** (0.009)	0.031*** (0.009)	0.031*** (0.009)	0.031*** (0.009)	0.031*** (0.009)	0.002 (0.008)	0.002 (0.008)	0.002 (0.008)	0.002 (0.008)	0.002 (0.008)
x4	−0.138** (0.064)	−0.134** (0.064)	−0.135** (0.064)	−0.137** (0.064)	−0.129** (0.064)	−0.100*** (0.033)	−0.099*** (0.033)	−0.100*** (0.033)	−0.101*** (0.033)	−0.096*** (0.033)
x5	0.011** (0.005)	0.011** (0.005)	0.011** (0.005)	0.011** (0.005)	0.011** (0.005)	−0.016*** (0.005)	−0.016*** (0.005)	−0.016*** (0.005)	−0.016*** (0.005)	−0.016*** (0.005)
x6	0.003 (0.003)	0.003 (0.003)	0.003 (0.003)	0.003 (0.003)	0.003 (0.003)	−0.007** (0.003)	−0.007* (0.003)	−0.007* (0.003)	−0.007** (0.003)	−0.006* (0.003)
x7	Control	Control	Control	Control	Control	Control	Control	Control	Control	Control
x8	Control	Control	Control	Control	Control	Control	Control	Control	Control	Control
x9	Control	Control	Control	Control	Control	Control	Control	Control	Control	Control
x10	Control	Control	Control	Control	Control	Control	Control	Control	Control	Control
x11	Control	Control	Control	Control	Control	Control	Control	Control	Control	Control
_cons	1.972*** (0.296)	1.987*** (0.297)	1.993*** (0.306)	1.944*** (0.301)	1.998*** (0.297)	2.247*** (0.230)	2.251*** (0.230)	2.301*** (0.230)	2.230*** (0.230)	2.207*** (0.230)
*R*2	0.096	0.096	0.096	0.096	0.096	0.076	0.076	0.076	0.076	0.077
*N*	90179.00	90179.00	90179.00	90179.00	90179.00	89409.00	89409.00	89409.00	89409.00	89409.00

(1) z1, z2, z3, z4 and z5 denote the number of individual students with a provincial fellow score rating of 1, 2, 3, 4 and 5 as a percentage of all fellows, respectively; Var denotes the variance of fellow scores. (2) x1, x2, x3, x4, x5, x6, x7, x8, x9, x10 and x11 indicate political affiliation, whether the student is poor, whether the student was born in an urban area, whether the student is a teacher-training student, parental education, help from parents and family background, province, school, major, type of club participation, and type of second degree minor subject. (3) Figures in parentheses are standard errors; (4) ***, ** and * represent 1, 5 and 10% significance levels respectively; (5) The above regression results are all ROBUST regressions.

In terms of the coefficient of the fellow effect, the coefficient of the fellow effect for male students tends to gradually increase, or the negative effect on male student’s individual grades decreases when the fellow grade is 1, 2, and 3 respectively, and the positive effect on male student’s individual grades increases when the fellow grade is 4 and 5, respectively. The coefficient of the fellow effect for female students tends to decrease and then increase, showing a U-shaped characteristic, or the negative effect on female students’ grades increases and then decreases when the fellow grade is 1, 2 and 3 respectively, and the positive effect on female students’ grades gradually increases when the fellow grade is 4 and 5, respectively. This means that male students are more likely to be negatively influenced by the lower academic performance of their peers, while female students are more likely to be positively influenced by the higher academic performance of their peers.

In terms of control variables, there was a significant positive effect of variance in fellow achievement on individual student achievement. Parental literacy had a significant positive effect on the academic achievement of individual male students, while it had a significant negative effect on the academic achievement of individual female students. The level of assistance from parents and family background did not have a significant effect on the academic performance of individual male students, while it had a significant negative effect on the academic performance of individual female students.

#### Regional differences in the fellow effect

This paper classifies the birthplaces of individual students into eastern, central, and western regions according to the China Statistical Yearbook. Table 5 shows the regression results of the fellow effect in the eastern, central and western regions, respectively. When the fellow ranking level is 1, 2 and 3 respectively, the fellow effect in the eastern and central regions has a significant negative effect on individual student’s performance, while the fellow effect in the western region is not significant; when the fellow ranking level is 5, it has a significant positive effect on individual students’ performance, and the fellow effect in each region The effect of fellow shows a decreasing trend from the eastern region to the central and western regions, i.e., east > central > west.

**Table 5:** Fellow effect in east, central and west regions.

The eastern region fellow effect						
Number of individual students with a provincial fellow score rating of i as a percentage of all peers		(1)	(2)	(3)	(4)	(5)
		Individual overall student performance ranking in the major				
	i = 1	−0.055 (0.071)				
	i = 2		−0.0871** (0.0433)			
	i = 3			−0.118*** (0.033)		
	i = 4				−0.019 (0.034)	
	i = 5					0.164*** (0.030)
The variance of provincial fellows’ results		0.027* (0.014)	0.026** (0.013)	0.017 (0.013)	0.022* (0.013)	0.020 (0.013)
_cons		2.851*** (0.130)	2.860*** (0.130)	2.882*** (0.130)	2.855*** (0.130)	2.783*** (0.130)
*R*2		0.128	0.128	0.129	0.128	0.130
*N*		20484.000	20484.000	20484.000	20484.000	20484.000
**The central region fellow effect**						
Number of individual students with a provincial fellow score rating of i as a percentage of all peers		(1)	(2)	(3)	(4)	(5)
		Individual overall student performance ranking in the major				
	i = 1	−0.207*** (0.066)				
	i = 2		−0.127*** (0.041)			
	i = 3			−0.074*** (0.028)		
	i = 4				0.080*** (0.029)	
	i = 5					0.085*** (0.025)
The variance of provincial fellows’ results		0.038*** (0.009)	0.032*** (0.009)	0.023*** (0.008)	0.032*** (0.009)	0.026*** (0.008)
_cons		3.058*** (0.257)	3.061*** (0.258)	3.083*** (0.268)	3.003*** (0.269)	3.062*** (0.258)
R2		0.092	0.092	0.092	0.092	0.092
N		142772.000	142772.000	142772.000	142772.000	142772.000
**The fellow effect in the west**						
Number of individual students with a provincial fellow score rating of i as a percentage of all peers		(1)	(2)	(3)	(4)	(5)
		Individual overall student performance ranking in the major				
	i = 1	−0.021 (0.079)				
	i = 2		−0.075 (0.049)			
	i = 3			−0.039 (0.036)		
	i = 4				0.018 (0.037)	
	i = 5					0.067* (0.035)
Variance of provincial fellows’ results		0.050*** (0.016)	0.051*** (0.015)	0.046*** (0.015)	0.050*** (0.015)	0.046*** (0.015)
_cons		2.784*** (0.193)	2.793*** (0.193)	2.796*** (0.194)	2.779*** (0.193)	2.764*** (0.193)
*R*2		0.110	0.110	0.110	0.110	0.110
*N*		16332.000	16332.000	16332.000	16332.000	16332.000

Control variables are consistent with Table 3.

In terms of the control variables, the variance of fellow achievement all had a significant positive effect on student achievement, and this effect declined from the west to the center and east. Gender had a significant negative effect on individual student achievement. Political affiliation, being a poor student, and being a teacher training student all had a positive effect on individual student achievement. Parental literacy and parental and family background assistance did not have a significant effect on individual student achievement. In the central and western regions, the effect of being born in an urban area was significantly positive, while in the eastern region, the effect of being born in an urban area was significantly negative.

#### Professional differences in the fellow effect

Table 6 shows the regression results of the fellow effect for humanities and social science majors, science and engineering majors. The effect of the fellow effect on individual student achievement is significantly negative when the overall fellow ranking rank is 1, 2 and 3 respectively, and the negative effect of the fellow effect is greater for humanities and social science majors than for science and technology majors. The effect of the fellow on individual student achievement is significantly positive for both humanities and social science majors when the ranking rank of fellow is 5, but the effect of fellow in humanities and social science majors is greater than that in science and engineering majors.

**Table 6:** fellow effect in humanities and social sciences, science and technology.

The fellow effect in humanities and social sciences						
Number of individual students with a provincial fellow score rating of i as a percentage of all peers		(1)	(2)	(3)	(4)	(5)
		Individual overall student performance ranking in the major				
	i = 1	−0.330*** (0.058)				
	i = 2		−0.359*** (0.038)			
	i = 3			−0.192*** (0.027)		
	i = 4				−0.019 (0.027)	
	i = 5					0.372*** (0.023)
Variance of provincial fellows’ results		0.008 (0.010)	0.003 (0.009)	−0.021** (0.009)	−0.014 (0.009)	−0.015* (0.009)
_cons		2.677*** (0.156)	2.721*** (0.155)	2.732*** (0.156)	2.675*** (0.155)	2.603*** (0.155)
*R*2		0.080	0.080	0.080	0.079	0.082
*N*		84142.000	84142.000	84142.000	84142.000	84142.000
**The fellow’s effect in science and engineering**						
Number of individual students with a provincial fellow score rating of i as a percentage of all peers	(1)		(2)	(3)	(4)	(5)
	Individual overall student performance ranking in the major					
	i = 1	−0.131** (0.055)				
	i = 2		−0.192*** (0.032)			
	i = 3			−0.185*** (0.024)		
	i = 4				0.018 (0.025)	
	i = 5					0.254*** (0.022)
Variance of provincial fellows’ results		−0.017* (0.009)	−0.018** (0.009)	−0.03 5*** (0.008)	−0.025*** (0.009)	−0.028*** (0.008)
_cons		2.642*** (0.127)	2.684*** (0.128)	2.715*** (0.127)	2.638*** (0.127)	2.599*** (0.127)
*R*2		0.097	0.097	0.097	0.097	0.098
*N*		95385.000	95385.000	95385.000	95385.000	95385.000

Control variables are consistent with Table 3.

In terms of control variables, the coefficients of influence for the variables of gender and whether or not they are teacher trainees are all significantly negative, while the coefficients of influence for political appearance and whether or not they are poor students are significantly positive. This is generally consistent with the above regression results.

### Robustness tests

To ensure the reliability of the findings, this paper uses municipal-level sample data to narrow the study of provincial-level fellow effects to municipal-level fellow effects. Consistent with the above, municipal-level fellows are located on a scale of 1–5 for this professional composite.

Table 7 shows the descriptive statistics for the municipal fellow data. After excluding the data of individual students without municipal fellows, the valid sample size was 149,206 entries. The maximum value of the overall grade ranking in the major is 5, the minimum value is 1 and the mean value is 3.675. The minimum value of the number of students with municipal fellow is 1, the maximum value is 802 and the standard deviation is 97.73. The core explanatory variables: the number of municipal fellows in different achievement levels as a percentage of all fellows, with means of 0.0044, 0.115, 0.275, 0.253 and 0.313 and standard deviations of 0.104, 0.160, 0.225, 0.217 and 0.244, respectively. in terms of individual student characteristics, the number of males and females was almost equal, the number of poor students, The number of students born in urban areas, and the number of teacher trainees were relatively small. The mean parental education level was 1.389, with a standard deviation of 0.816, indicating that the majority of students’ parents had a low level of education, mainly at the junior to senior secondary level. The level of parental and family background assistance to students in all areas is low, with a mean value of 1.945.

**Table 7:** Descriptive statistics (municipal).

Main variables	Sample size	Average	Minimum value	Maximum value	Standard deviation
Overall ranking in the program	149,206	3.675	1	5	1.158
Number of individual students’ municipal fellows	149,206	41.70	1	802	97.73
Individual student’s municipal fellow score rating of 1 as a percentage of all fellows	149,206	0.0440	0	1	0.104
Individual student’s municipal fellow score rating of 2 as a percentage of all fellows	149,206	0.115	0	1	0.160
Individual student’s municipal fellow score rating of 3 as a percentage of all fellows	149,206	0.275	0	1	0.225
Individual student’s municipal fellow score rating of 4 as a percentage of all fellows	149,206	0.253	0	1	0.217
Individual student’s municipal fellow score rating of 5 as a percentage of all fellows	149,206	0.313	0	1	0.244
Variance of municipal fellows’ results	149,206	0.972	0	4	0.644
Gender	149,206	0.501	0	1	0.500
Whether you are a needy student	149,206	0.138	0	1	0.344
Born in an urban area or not	149,206	0.254	0	1	0.435
Political affiliation	149,206	0.968	0	3	0.398
Second Degree Minor Subject Categories	149,206	0.685	0	13	2.349
Type of association attended	149,206	4.287	0	11	3.278
Parental literacy	149,206	1.389	0	5	0.816
Help from parents and family background	149,206	1.945	1	5	1.130
Whether a student majoring in normal education	149,206	0.113	0	1	0.316

The results of the robustness tests in Table 8 show that there is a significant negative effect of the proportion of municipal fellow on individual students’ overall grades when the municipal fellow achievement level is 2 and 3 respectively; when the municipal fellow achievement level is 5, there is a significant positive effect of the proportion of municipal fellow on individual student’s overall grade, which is consistent with the regression results of the effect of the provincial fellow effect on individual student’s overall grade in Table 3 above This is consistent with the results of the regression of the effect of a provincial level fellow on individual students’ overall grades in Table 3 above, further confirming the existence of the fellow effect.

**Table 8:** Robustness tests.

		(1)	(2)	(3)	(4)	(5)
		Individual overall student performance ranking in the major				
Number of individual students with a municipal fellow score rating of i as a percentage of all fellows	i = 1	−0.005 (0.031)				
	i = 2		−0.079*** (0.019)			
	i = 3			−0.075*** (0.013)		
	i = 4				0.010 (0.014)	
	i = 5					0.091*** (0.012)
Variance of municipal fellows’ results		−0.010* (0.006)	−0.007 (0.005)	−0.015*** (0.005)	−0.010* (0.005)	−0.012** (0.005)
Gender		−0.458*** (0.008)	−0.458*** (0.008)	−0.458*** (0.008)	−0.458*** (0.008)	−0.458*** (0.008)
Political affiliation		0.375*** (0.007)	0.375*** (0.007)	0.374*** (0.007)	0.375*** (0.007)	0.374*** (0.007)
Whether you are a needy student		0.231*** (0.009)	0.231*** (0.009)	0.231*** (0.009)	0.231*** (0.009)	0.231*** (0.009)
Born in an urban area or not		0.005 (0.007)	0.005 (0.007)	0.005 (0.007)	0.005 (0.007)	0.005 (0.007)
Whether a student majoring in normal education		−0.091*** (0.033)	−0.088*** (0.033)	−0.089*** (0.033)	−0.090*** (0.033)	−0.088*** (0.033)
Parental literacy		−0.001 (0.004)	−0.000 (0.004)	−0.000 (0.004)	−0.001 (0.004)	−0.000 (0.004)
Help from parents and family background		−0.000 (0.003)	−0.000 (0.003)	−0.000 (0.003)	−0.000 (0.003)	0.000 (0.003)
Province		Control	Control	Control	Control	Control
School		Control	Control	Control	Control	Control
Specialties		Control	Control	Control	Control	Control
Type of association attended		Control	Control	Control	Control	Control
Second degree minor subject categories		Control	Control	Control	Control	Control
_cons		2.120*** (0.342)	2.138*** (0.343)	2.162*** (0.365)	2.116*** (0.346)	2.143*** (0.342)
R2		0.095	0.095	0.096	0.095	0.096
N		149206.000	149206.000	149206.000	149206.000	149206.000

(1) Figures in brackets are standard errors; (2) ***, ** and * represent 1, 5, and 10% significance levels respectively; (3) The above regression results are all ROBUST regressions.

Compared to the results in Table 3, the coefficient on the proportion of municipal fellows on individual student achievement levels is not significant at a municipal fellow achievement level of 1, and the coefficients on all five core explanatory variables are reduced for two possible reasons: first, the number of individual students with municipal fellows is much smaller than the number of fellows at the provincial level, so the effect of municipal fellows is smaller than the effect of provincial fellows. Secondly, the differences in language, culture, lifestyle, values, religious practices, economic policies, and educational policies between cities in a province are small, whereas these factors vary considerably between provinces, which may also lead to a smaller effect on fellow at the municipal level than at the provincial level.

### Endogeneity test

To address the imaging problem in the impact of the fellow effect on individual student academic performance, the average literacy of provincial old-town parents was selected as an instrumental variable in this paper. We used two-stage least squares with instrumental variables to develop the following model.


(2) $$\mathrm{Phase}\;I:Z_{ijp}=fellow\_parent\_edu_{ijp}+X_{control}+u_{i}$$

(3) $$\mathrm{Phase}\;\mathrm{II}:Y_{ijk}=Z_{ijp}+X_{control}+\varepsilon_{i}$$

$Y_{ijk},Z_{ijp}$ and$X_{control}$ have the same meaning as in eq. (1), and$fellow\_parent\_edu_{ijp}$ denotes the average educational attainment of the parents of provincial fellows. Since the average educational attainment of the parents of provincial fellows affects the performance of the fellows in terms of academic achievement, it does not have an impact on the academic achievement of individual students. Therefore, the $\;COV\left(Z_{ijp,}fellow\_parent\_edu_{ijp}\right)\neq 0$, the$\;COV\left(Y_{jik},fellow\_parent\_edu_{ijp}\right)=0$, the instrumental variables satisfy both correlation and exogeneity. In the first stage, the core explanatory variables were first used$\;\left(Z_{ijp}\right)$ for the average educational attainment of the parents of the fellows ($fellow\_parent\_edu_{ijp}$) was estimated by regression. In the second stage, the fitted core explanatory variables were estimated using the individual student’s overall performance ranking $\left(Z_{ijp}\right)$ regression estimation was conducted.

Table 9 reports the results of the regressions using instrumental variables and the results of the tests for the validity of the instrumental variables. The results show that: (1) the first stage regression results for the number of fellows as a percentage of all fellows are significant when the individual student’s provincial fellow grades are 1, 2, and 3, respectively, and pass the under-identification test and the weak instrumental variable test; the second stage regression results show that the fellow effect has a significant negative impact on the individual student’s academic performance. (2) The first-stage regression results were significant when the individual students’ provincial fellow grades were 4 and 5 respectively, and passed the under-identification test and the weak instrumental variable test; the second-stage regression results showed that the fellow effect had a significant positive impact on the individual student’s academic performance.

**Table 9:** Two-stage regression results for instrumental variables.

			Explained variables				
Second-stage regression results	Explanatory variables		Individual overall student performance ranking in the major				
	Number of individual students with a provincial fellow score rating of i as a percentage of all peers	i = 1	−10.887 (7.001)				
		i = 2		−2.274* (1.389)			
		i = 3			−2.160* (1.196)		
		i = 4				2.338* (1.304)	
		i = 5					1.764* (0.968)
First-stage regression results			Explained variables				
	Explanatory variables		Number of individual students with a provincial fellow score rating of i as a percentage of all fellows				
			i = 1	i = 2	i = 3	i = 4	i = 5
	The average literacy level of parents in the provincial fellow		0.001*** (0.00049)	0.006*** (0.00079)	0.007*** (0 0.00105)	−0.0063*** (0.00102)	−0.008*** (0.00115)
Under-identification test	LM statistic		7.559	69.122	42.845	38.564	53.127
	value of p		0.006	0.000	0.000	0.000	0.000
Weak instrumental variables test	Cragg-Donald Wald F statistic		7.526	68.838	42.663	38.398	52.904
	Stock-Yogo bias critical values (15%)		8.96	8.96	8.96	8.96	8.96

Control variables are consistent with OLS regression results.

## Further discussion

In some of the regression results in Tables 4, 5, as well as in Table 6 for the effect of fellow in humanities and social sciences and science and technology, and in Table 8 for the robustness test, when the core explanatory variable is the number of fellow students with a provincial fellow achievement grade of 4 as a percentage of all fellow students, the effect of this variable on individual student academic achievement is not significant, possibly because the mean of the overall achievement rank of individual students in the sample is located in the major of 3.682, could this indicate that fellow with an achievement grade of 4 would not have an impact on individual students with an achievement grade close to that mean.

According to the regression results in Table 10, the effect of the number of students with a provincial fellow grade of 4 as a percentage of all fellows on individual students’ academic performance changed from a non-significant to a significant positive effect, while the effect of the number of students with a provincial fellow grade of 3 as a percentage of all fellows on individual students’ academic performance changed from a significant negative to a non-significant effect. The effect of the number of students with a provincial fellow score of 3 as a percentage of all fellows on individual students’ academic performance changed from a negative to a non-significant effect. Therefore, this paper concludes that the effect of fellows on students’ academic performance is not significant when the gap between students’ academic performance and that of their fellows is small, and that the effect of fellows on students’ academic performance is significant only when the gap between students’ academic performance and that of their fellow is large.

**Table 10:** Regression results after adjusting for mean values.

		(1)	(2)	(3)	(4)	(5)
		Individual overall student performance ranking in the major				
Number of individual students with a provincial fellow score rating of i as a percentage of all peers	i = 1	−0.056* (0.032)				
	i = 2		−0.086*** (0.022)			
	i = 3			−0.022 (0.017)		
	i = 4				0.039* (0.022)	
	i = 5					0.097*** (0.021)
Gender		−0.392*** (0.009)	−0.393*** (0.009)	−0.392*** (0.009)	−0.392*** (0.009)	−0.392*** (0.009)
Political affiliation		0.323*** (0.010)	0.323*** (0.010)	0.324*** (0.010)	0.323*** (0.010)	0.323*** (0.010)
Whether you are a needy student		0.167*** (0.011)	0.167*** (0.011)	0.167*** (0.011)	0.167*** (0.011)	0.166*** (0.011)
Born in an urban area or not		0.047*** (0.008)	0.047*** (0.008)	0.047*** (0.008)	0.047*** (0.008)	0.047*** (0.008)
Whether a student majoring in normal education		−0.084** (0.037)	−0.082** (0.037)	−0.085** (0.037)	−0.084** (0.037)	−0.081** (0.037)
Parental literacy		0.002 (0.004)	0.002 (0.004)	0.002 (0.004)	0.002 (0.004)	0.002 (0.004)
Help from parents and family background		0.000 (0.003)	0.000 (0.003)	0.001 (0.003)	0.000 (0.003)	0.001 (0.003)
Province		Control	Control	Control	Control	Control
School		Control	Control	Control	Control	Control
Specialties		Control	Control	Control	Control	Control
Type of association attended		Control	Control	Control	Control	Control
Second degree minor subject categories		Control	Control	Control	Control	Control
_cons		2.598*** (0.114)	2.603*** (0.114)	2.593*** (0.114)	2.556*** (0.114)	2.592*** (0.114)
*R*2		0.087	0.088	0.087	0.087	0.088
*N*		95,757	95,757	95,757	95,757	95,757

(1) Figures in brackets are standard errors; (2) ***, ** and * represent 1, 5, and 10% significance levels respectively; (3) the above regression results are all ROBUST regressions.

## Conclusion and discussion

While peer groups in schools have significant effects on adolescent development, the influence of fellow groups, which “carry” common perceptions, cultural dispositions, and regional psychology, on adolescents in schools has not been well studied. This paper examines the effect of fellows on the academic performance of college students based on an OLS model using data from the 2018 college graduate employment survey in a central province. The study found that:

1. There is a significant fellow effect on the academic achievement of college students. The fellow with grade ranking in the middle and lower reaches of their major have a significant negative effect on the academic performance of individual students, while fellows with grade ranking in the upper reaches of their major have a significant positive effect on the academic performance of individual students.
2. There are gender differences in the effect of fellow. The coefficient of the effect of different academic grades of a fellow on male students’ academic performance tends to gradually increase, while the coefficient of the effect of different academic grades of a fellow on female students’ academic performance shows a U-shaped characteristic of first decreasing and then increasing. Male students are more likely to be negatively influenced by fellows with poorer academic performance; while females are more significantly influenced by fellows with better academic performance. This may be due to the fact that female students have a stronger sense of learning and study harder and more solidly, while male students are more active and more susceptible to peer interference, resulting in gender differences in the fellow effect.
3. The fellow effect not only has gender differences but also regional differences, with the positive effect of fellow achievement on individual students’ performance gradually decreasing from the eastern to the central and western regions. One of the reasons for the regional differences in the fellow effect may be that the level of education in each region gradually decreases from the eastern to the central and western regions, resulting in significant differences in students’ perceptions and attitudes toward learning in each region.
4. Regarding the fellow effect between different professional categories, the negative effect of fellow performance on students’ individual performance is greater in humanities and social science majors than in science and engineering majors; in terms of the positive effect of fellow performance on students’ individual performance, the fellow effect is greater in humanities and social science majors than in science and engineering majors. This may be due to the fact that science and engineering majors focus on rational analysis and logical deduction, while humanities and social science majors focus more on comprehension and memorization, and summarization, which leads to the difference in the fellow effect among different major categories.

There may be several shortcomings in this paper: Firstly, in the regressions grouped by gender, this paper only examines the effect of all male (female) fellows on the academic performance of individual male (female) students and does not consider the effect of heterosexual fellows on the academic performance of individual students. Secondly, this paper uses cross-sectional data, which does not allow for dynamic analysis of the effect of fellow on individual students across grades. Third, few scholars have studied the fellow effect, so this paper lacks appropriate theoretical support.

## Data availability statement

The original contributions presented in the study are included in the article/supplementary material, further inquiries can be directed to the corresponding author.

## Author contributions

GZ: conception, data collection, analysis, revision. MZ: writing and literature review, empirical analysis, revision. WW: revision, fund support. All authors contributed to the article and approved the submitted version.

## Funding

This study was financially supported by the National Social Science Foundation of China (nos. 21XGL02 and 18BGL246), the Youth Jinggang Scholars Program in Jiangxi Province (no. QNJG2020047), the Jiangxi Higher Education Teaching Reform Research Project (no. JXJG-20-7-32), the Basic Research Plan of The Science and Technology Plan Project of Jiangxi Provincial Science and Technology Department (no. 20181BAA208005) and the Key Project of Jiangxi Education Planning (no. 17ZD027).

## Conflict of interest

The authors declare that the research was conducted in the absence of any commercial or financial relationships that could be construed as a potential conflict of interest.

## Publisher’s note

All claims expressed in this article are solely those of the authors and do not necessarily represent those of their affiliated organizations, or those of the publisher, the editors and the reviewers. Any product that may be evaluated in this article, or claim that may be made by its manufacturer, is not guaranteed or endorsed by the publisher.

## References

[ref1] Bandura A. (2001). The Social Foundations of Thought and Action: A Theory of Social Cognition. Shanghai: East China University Press.

[ref2] Bramoullé Y. Djebbari H. Fortin B. (2020). Peer effects in networks: a survey. Ann. Rev. Econ. 12, 603–629. doi: 10.1146/annurev-economics-020320-033926

[ref3] Bruce S. (2001). Peer effects with random assignment: results for Dartmouth roommates. Q. J. Econ. 2, 681–704. doi: 10.1162/00335530151144131

[ref4] Caiting D. Yuanyuan C. (2021). Peer effects of adolescents' electronic media use – a perspective based on classroom social networks[J]. Financ. Res. 47, 125–139. doi: 10.16538/j.cnki.jfe.20210313.302

[ref5] Coleman J. S. Equality of Educational Opportunity. Washington: U.S. Govt, 1966.

[ref6] Diemer A. (2022). Endogenous peer effects in diverse friendship networks: evidence from Swedish classrooms. Econ. Educ. Rev. 89:102269. doi: 10.1016/j.econedurev.2022.102269

[ref7] Eisenberg D. Golberstein E. Whitlock J. L. (2014). Peer Effects on Risky Behaviors. J. Health Econ. 33, 126–138. 24316458 10.1016/j.jhealeco.2013.11.006

[ref8] Guo S. P. Zhang X. B. (2022). Re-evaluation of Bandura's social learning theory from a cultural psychology perspective. Psychol. Res. 15, 99–104. doi: 10.19988/j.cnki.issn.2095-1159.2022.02.001

[ref1001] Isabel Pessoa de Arruda Raposo Gonalves M. B. C. (2018). *Peer effects and scholastic achievement: Evidence of causal effects using age at school entry as exogenous variation for peer quality*. Available at: https://econpapers.repec.org/paper/anpen2016/209.htm

[ref9] Jiang Y. Z. Zhang H. Z. Zhang P. Y. (2012). A theoretical exploration and empirical study of the psychological effects of old folks in China. Adv. Psychol. Sci. 20, 1237–1242. doi: 10.3724/SP.J.1042.2012.01237

[ref10] Kelley H. H. Two Functions of Reference Groups. New York: Henry Holt & Co, 1952. 410–414.

[ref11] Liu Y. Ma Z. (2022). Does the fellow relationship between CEO and directors affect corporate financial risk? A study on the causes of financial risk based on factor analysis construction. Finance Account. Commun. 8, 29–34+117. doi: 10.16144/j.cnki.issn1002-8072.2022.08.015

[ref12] Ma L.-P. Huang Y.-F. (2021). "Close to the vermilion" or "exclusive competition" -a study of roommate peer effects on academic development of elite university students. Beijing Univ. Educ. Rev. 19, 41–63+189. doi: 10.12088/pku1671-9468.202102003

[ref13] Manski C. F. (1993). Identification of endogenous social effects: the reflection problem. Rev. Econ. Stud. 60, 531–542. doi: 10.2307/2298123

[ref14] Qiang L. (2019). The impact of peer effects on children dropping out of compulsory education in rural areas. Educ. Econ. 4, 36–44. doi: 10.3969/j.issn.1003-4870.2019.04.005

[ref15] Raposo I. Gonalves M. (2018). Peer Effects and Scholastic Achievement: Evidence of Causal Effects Using Age at School Entry as Exogenous Variation for Peer Quality//Anais do XLIV Encontro Nacional de Economia [Proceedings of the 44th Brazilian Economics Meeting]. ANPEC – AssociaÃ§Ã£o Nacional dos Centros de PÃ3s-GraduaÃ§Ã£o em Economia [Brazilian Association of Graduate Programs in Economics].

[ref16] Sacerdote B. (2011). Peer effects in education: how might they work, how big are they and how much do we know thus far? Handb. Econ. Educ. 3, 249–277. doi: 10.1016/B978-0-444-53429-3.00004-1

[ref17] Shigen Z. (2019). "fellow" relationship between CEOs and directors, internal control quality and agency costs. Financ. Theory Pract. 40, 88–94. doi: 10.16339/j.cnki.hdxbcjb.2019.04.036

[ref18] Wu G. Zhang F. (2020). The health costs of being "close to the vermilion": peer influence and adolescents' academic performance and mental health. Educ. Res. 41, 123–142. doi: 10.37155/2717-5650-0105-8

[ref19] Xiaojuan Q. (2015). A study of peer influence on college performance: an analysis based on a multilevel model. Tsinghua Univ. Educ. Res. 36, 66–76. doi: 10.14138/j.1001-4519.2015.05.006611

[ref20] Xuesong W. (2020). The influence of housemates on college students' academic performance – a peer effect based on randomly assigned dormitories. Rev. Educ. Econ. 5, 77–90. doi: 10.19512/j.cnki.issn2096-2088.2020.06.005

[ref21] Yang Z. Huang B. (2020). Close to the vermilion: do good peers have a positive influence? Rev. Educ. Econ. 5, 108–125. doi: 10.19512/j.cnki.issn2096-2088.2020.03.007

[ref22] Yaoming L. Qinying H. (2017). Analysis of dormitory peer effects on college students' academic performance. Educ. Econ. 4, 83–88.

[ref23] Zhang H. Z. Jiang Y. Z. (2010). The psychological representation of Chinese people's perception of fellow and its psychological mechanism. J. Liaoning Normal Univ. (Soc. Sci. Ed.) 33, 42–45. doi: 10.3724/SEJ.1042.2012.01237

[ref24] Zhang H. Z. Jiang Y. Z. (2013). A study on the psychological effects of Gansu people's fellow and its influencing factors. J. Chongqing Coll. Arts Sci. 32, 134–138. doi: 10.19493/j.cnki.issn1673-8004.2013.04.031

[ref25] Zhimei B. (2020). A study on the peer effect of undergraduate students' academic performance. China High. Educ. Res. 6, 25–31. doi: 10.16298/j.cnki.1004-3667.2020.06.06

[ref26] Zhong Z. H. Qian Y. (2017). Identification and analysis of the psychological effects of fellow association hall and fellow and social management strategies. J. Xinyi Natl Teach. Coll. 2, 8–11. doi: 10.3969/j.issn.1009-0673.2017.02.003

[ref27] Zhouhang Y. Shi M. Cheng X. (2018). The effect of peer effects on academic performance in rural primary schools: is it better to be close to the vermilion? Educ. Econ. 1, 65–73. doi: 10.3969/j.issn.1003-4870.2018.01.013

